# Identifying key genes and screening therapeutic agents associated with diabetes mellitus and HCV-related hepatocellular carcinoma by bioinformatics analysis

**DOI:** 10.1016/j.sjbs.2021.07.068

**Published:** 2021-07-30

**Authors:** Muhammad Sufyan, Usman Ali Ashfaq, Sajjad Ahmad, Fatima Noor, Muhammad Hamzah Saleem, Muhammad Farhan Aslam, Hamed A. El-Serehy, Sidra Aslam

**Affiliations:** a Department of Bioinformatics and Biotechnology, Government College University Faisalabad (GCUF), Allama Iqbal Road, Faisalabad-38000, Pakistan; b Department of Health and Biological Sciences, Abasyn University, Peshawar, Pakistan; c MOA Key Laboratory of Crop Ecophysiology and Farming System in the Middle Reaches of the Yangtze River, College of Plant Science and Technology, Huazhong Agricultural University, Wuhan 430070, China; d School of Biological Sciences, University of Edinburgh, United Kingdom; e Department of Zoology, King Saud University, Riyadh 11451, Saudi Arabia

**Keywords:** Bioinformatics analysis, Hepatocellular carcinoma, Type 2 diabetes mellitus, Gene expression profiling, Hub genes, Candidate drugs, MCOD, Molecular Complex Detection, HCC, Hepatocellular Carcinoma, T2DM, Type2 Diabetes Mellitus, PPI, Protein-Protein Interaction network, GO, Gene Ontology, DEGs, Differential Expressed Genes, TFs, Transcription factors

## Abstract

**Objective:**

Incidence of both Type 2 diabetes mellitus (T2DM) and hepatocellular carcinoma (HCC) are rapidly increasing worldwide. One of the leading causes of HCC is hepatitis C virus (HCV), which is a resource of blood-borne viral infection. HCV increases the risk for HCC probably by promoting fibrosis and cirrhosis. Association among T2DM and HCV related HCC remains significant, indicating that such association is clinically reliable and robust. Lawson was the first who uncovered HCC in person suffered from T2DM. Until now, genetic association between HCV related HCC and T2DM is poorly known. Current work was designed to figure out the molecular mechanisms of both diseases by identifying the hub genes and therapeutic drugs using integrated bioinformatics analysis.

**Methods:**

Four microarray datasets were downloaded from GEO database and analyzed using R in order to obtain different expressed genes (DEGs). Protein–protein interaction (PPI) networks was constructed using STRING tool and visualized by Cytoscape. Moreover, hub genes were identified on the basis of their degree of connectivity. Finally, Networkanalyst and DGIdb were used for the identification of transcription factors (TFs) and selection of candidate drugs, respectively.

**Results:**

A total of 53 DEGs were identified, of which 41 were upregulated genes and 12 were downregulated genes. PPI network obtained from STRING were subjected to Cytoscape plugin cytoHubba, and top 10 genes (AURKA, JUN, AR, MELK, NCOA2, CENPF, NCAPG, PCK1, RAD51AP1, and GTSE1) were chosen as the target hub genes based on the highest degree of connectivity. Furthermore, 47 drugs of AURKA, JUN, AR, MELK, and NCOA2 were found having therapeutic potential to treat HCV-HCC in patients with T2DM.

**Conclusion:**

This study updates the information and yield a new perspective in context of understanding the pathogenesis and development of HCV related HCC in affected persons with T2DM. *In vivo* and *in vitro* investigation of hub genes and pathway interaction is essential to delineate the specific roles of the novel hub genes, which may help to reveal the genetic association between HCV-HCC and T2DM. In future, hub genes along with their candidate drugs might be capable of improving the personalized detection and therapies for both diseases.

## Graphical abstract

**Figure:**
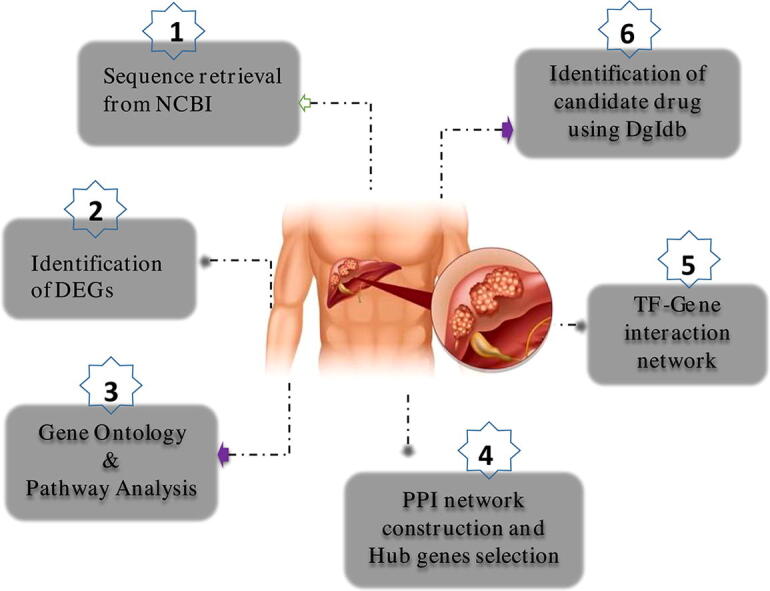

## 1 Introduction

Type 2 diabetes mellitus (T2DM) is the third main chronic metabolic disorder, which threatens public health worldwide. It is a complex multifactorial disease caused by environmental and genetic factors (Chatterjee et al., 2017, Dong et al., 2019). T2DM is characterized by insulin deficiency, and hyperglycemia. However, it is not only related to cardiovascular and nephropathy diseases but also related to several liver diseases (Olokoba et al., 2012). From 2015, it was found that about 90.5% of diabetes cases is a result of diabetes mellitus type II and is more prevalent in under-developing and developing countries presenting more risk to certain ethnic groups at global level (Leahy, 2005, Sharma et al., 2016, Chen et al., 2012). Moreover, it is more frequently diagnosed in children with high rate of obesity across the world (Zheng et al., 2018).

The most common and primary type of liver cancer is hepatocellular carcinoma (HCC) which usually occurs in people having acute diseased conditions such as cirrhosis, hepatitis A or C (di Bisceglie et al., 1988, McGlynn et al., 2021, Bréchot et al., 2000, Ozakyol, 2017). One of the leading causes of HCC is hepatitis C virus (HCV) which is a resource of blood-borne viral infection (de Oliveria Andrade et al., 2009). Slew of studies proved a strong association among HCC and HCV. HCV increases the risk for HCC probably by promoting fibrosis and cirrhosis; virtually all HCV-related HCC cases occur among patients with cirrhosis (El-Serag, 2012).

In 1987, Lawson elaborated a relation in between HCC and DM which is the sixth most common type of cancer cell occurring in human’s accounting about 12% of human death (El-Serag et al., 2004). Observational studies from Asia, Europe and America supported the fact that there exists a relationship in between DM and HCC risk factors presented as independent factors (El–Serag et al., 2006). It is related to increased proliferation effects as in case of hyperglycemia and inflammatory effects of obesity. Diabetes has proved to be condition and is associated with various types of malignancies such as increased occurrence of non-fatty liger diseases (Lawson et al., 1986). So, hepatitis C infection, consumption of alcohol and diabetes mellitus are strong etiologic factors for HCC in future (Hassan et al., 2002). Hence there exists a strong positive relationship between T2DM and development of risk associated with HCC (Donadon et al., 2008).

The discovery of potential biomarkers that can halt the pathophysiology of the disease and can act as a virtual shortcut, will considered as the miracle of the current era. Mind boggling potential benefits of molecular biomarkers offers multiple innovative perspective to improve diagnostic as well as treatment option. Now a days, the use of bioinformatics is getting popular across all facets of life sciences. Recently, it has been seen as an outbreak of emerging sequencing technologies that enable researchers to make ground-breaking discoveries in the domain of computational biology. In recent decades, bioinformatics along with microarray technologies has paved the way of researchers to identify disease related genes involved in the pathogenesis of HCC in persons with T2DM. Various bioinformatics related researches on plenty of human diseases had proven reliable and persuasive, so it implies that integrated bioinformatics analysis can contribute to evaluate the complex molecular mechanism underlying the development of HCC in patient suffering from T2DM.

No study is conducted yet to find the genetic association between HCV related HCC and T2DM, even that no sufficient evidence is present yet to prove the existence of disease related genes, and their involvement in the pathogenesis and development of HCV-HCC in persons with T2DM. To tackle this issue, we conducted integrated bioinformatics approaches to figure out the disease related functional genes as problem-solving negotiators to switch off the progression of both diseases. Moreover, investigation of drug-genes interactions in the present work can contribute to the discovery of therapeutic candidates for drug repurposing.

## 2 Materials and methods

### 2.1 Data sources

NCBI-GEO database is publicly accessible database that contains gene expression datasets (Barrett et al., 2005). Three gene expression profile datasets of HCV related hepatocellular carcinoma (GSE62232, GSE69715, GSE107170) and one dataset of T2DM (GSE15653) were retrieved from GEO database (https://www.ncbi.nlm.nih.gov/geo/). Detailed information of microarray datasets was provided in Table 1. Gene expression profiles were set accordingly such as (1) Tissues samples collected from diseased liver tissues and normal liver tissues, (2) number of samples were obtained for each dataset were more than 3.

**Table 1:** List of datasets used in this study.

**Datasets**	**Disease**	**Platform**	**Control**	**Affected**	**sample**
GSE62232	HCV related hepatocellular carcinoma	GPL570	10	9	Liver
GSE69715	HCV related hepatocellular carcinoma	GPL570	66	37	Liver
GSE107170	HCV related hepatocellular carcinoma	GPL570	31	44	Liver
GSE15653	Diabetes mellitus	GPL96	4	4	Liver

### 2.2 Identification of Differential Expressed genes (DEGs)

Differentially Expressed Genes (DEGs) for both diseases were identified separately using the NCBI- GEO2R, which is an interactive tool used to analyze and compare the data of two or more different sample groups under the similar experimental conditions (Barrett et al., 2012). Genes that satisfy the criteria of |log fold change (FC)| > 1.0 and adjusted P-value < 0.05 were distinguished as DEGs. Genes showing up-regulation or down-regulation in both HCC-HCV and T2DM were identified by the Venn diagram web tool (http://bioinfogp.cnb.csic.es/tools/venny/).

### 2.3 Analysis of DEGs at functional level

Gene Ontology (GO) enrichment analysis and KEGG pathways analysis were conducted for the predictions of impending functions of the hub genes using the DAVID (Database for Annotation, Visualization, and Integrated Discovery) online tool (Sherman et al., 2007). DEGs were subjected to DAVID for the prediction of the function of DEGs at three level: Molecular function (MF), Biological process (BP), and Cellular component (CC). Bubble maps were generated for the CC, MF, BP and KEGG pathways by employing the ggPlot2 (R package) based on statistically significant P-value (P < 0.05).

### 2.4 PPI network construction and hub genes identification

Interactive network of the common DEGs among all datasets were constructed through an online interactive search tool STRING (https://string-db.org/) with interactions score > 0.5. PPI network was further visualized and analyzed through the Cytoscape version 3.8.2 (Demchak et al., 2014). Molecular Complex Detection (MCODE) plugin from Cytoscape was utilized for distinguishing the module that best represent the clusters of DEGs. In MCODE, the modules were considered significant having number of nodes ≥ 3 and the score ≥ 3. Further, the resulted three modules were subjected to DAVID for the KEGG pathway analysis. Lastly, Top 10 hub genes among DEGs were identified using the Cytohubba plugin in Cytoscape based on highest degree of connectivity.

### 2.5 TF-Gene interaction network

Networkanalyst database was used to explore the human transcription factors (TFs) of the related hub genes (Xia et al., 2014). Networkanalyst database integrate three databases named JASPAR, ENCODE and ChEA. In the current analysis, ChIP Enrichment Analysis (ChEA) database was used to find the target TFs of hub genes (Xia et al., 2015). Moreover, Cytoscape tool was used to visualize the interaction network among TFs and hub genes.

### 2.6 Drug-gene interaction

Using Drug gene interaction database (DGIdb), drugs were chosen based on hub genes that acted as enthralling and promising target (Griffith et al., 2013). Only drugs that had been approved by the Food and Drug Administration and having DrugBank source were included in this final drug list.

## 3 Results

### 3.1 Identification of DEGs

In the present study, four microarray datasets (GSE62232, GSE69715, GSE107170, and GSE15653) were obtained from GEO database and found the DEGs using GEO2R tool. The resulted DEGs of each dataset were subjected to Venn diagrams for the identification of overlapped genes among four microarray datasets. A total of 53 overlapped genes were identified, 41 upregulated and 12 downregulated genes (Table 2).

**Table 2:** A total 53 DEGs were identified of which 41 were upregulated and 12 were downregulated genes.

**Differential Expressed Genes (DEGs)**	**Name of the genes**
**Upregulated genes** **(41)**	TPR, SQLE, SPINK1, MELK, CENPF, NCAPG, AURKA, DTNA, TRPM3, CLGN, IGF2BP3, SULT4A1, F5, RUNX1, BAX, PDCD2, GALNT10, SSR3, RCN2, RABIF, RAD51AP1, LUC7L3, GPD2, TPGS2, NCOA2, GTSE1, GPX2, NUDT3, CD58, DLAT, ZBTB38, SMAD5, MLEC, HIC2, CXCL11, GNAL, FADS1, KDM5B, POGZ, AR, CYP3A4
**Downregulated genes** **(12)**	PCK1, DUSP1, ALDOB, SPATA6, JUN, SGCD, TM4SF1, MNDA, PLIN2, CXCL2, IGFBP1, LRRC8B

### 3.2 Analysis of DEGs at functional level

GO enrichment and KEGG pathways analysis of DEGs were performed to analyze the gene function in terms of biological processes, cellular components, and molecular function as well as their associated pathways. GO enrichment analysis of top 10 significantly enriched terms showed that in BP category, the genes involved are concerned with negative regulation of transcription, DNA-templated, aging, oxidation–reduction process, positive regulation of apoptotic process, cell division, and cell–cell signaling. In terms of CC, the genes were enriched in nuclear envelope, endoplasmic reticulum membrane, nucleus, pronucleus, and cytosol. For MF, category the genes were mainly concentrated in the transcription factor binding, enzyme binding, protein binding, and chromatin binding. KEGG enrichment pathway analysis revealed that genes were significantly enriched in glycolysis/gluconeogenesis, biosynthesis of antibiotics, and pathways in cancer.

### 3.3 Construction of PPI network and the analysis of DEGs

**Fig. 1:**
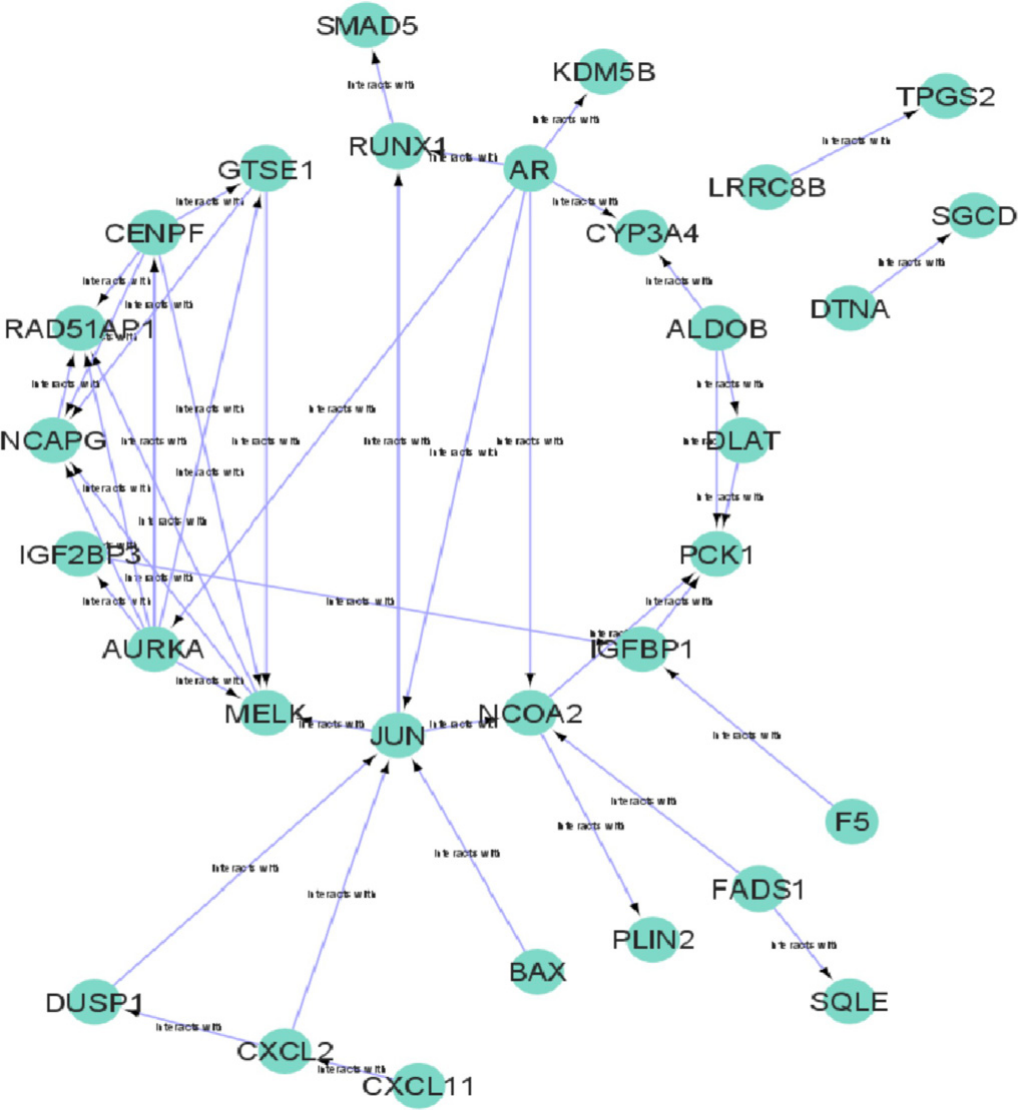
PPI network of 53 commonly identified DEGs.

**Fig. 2:**
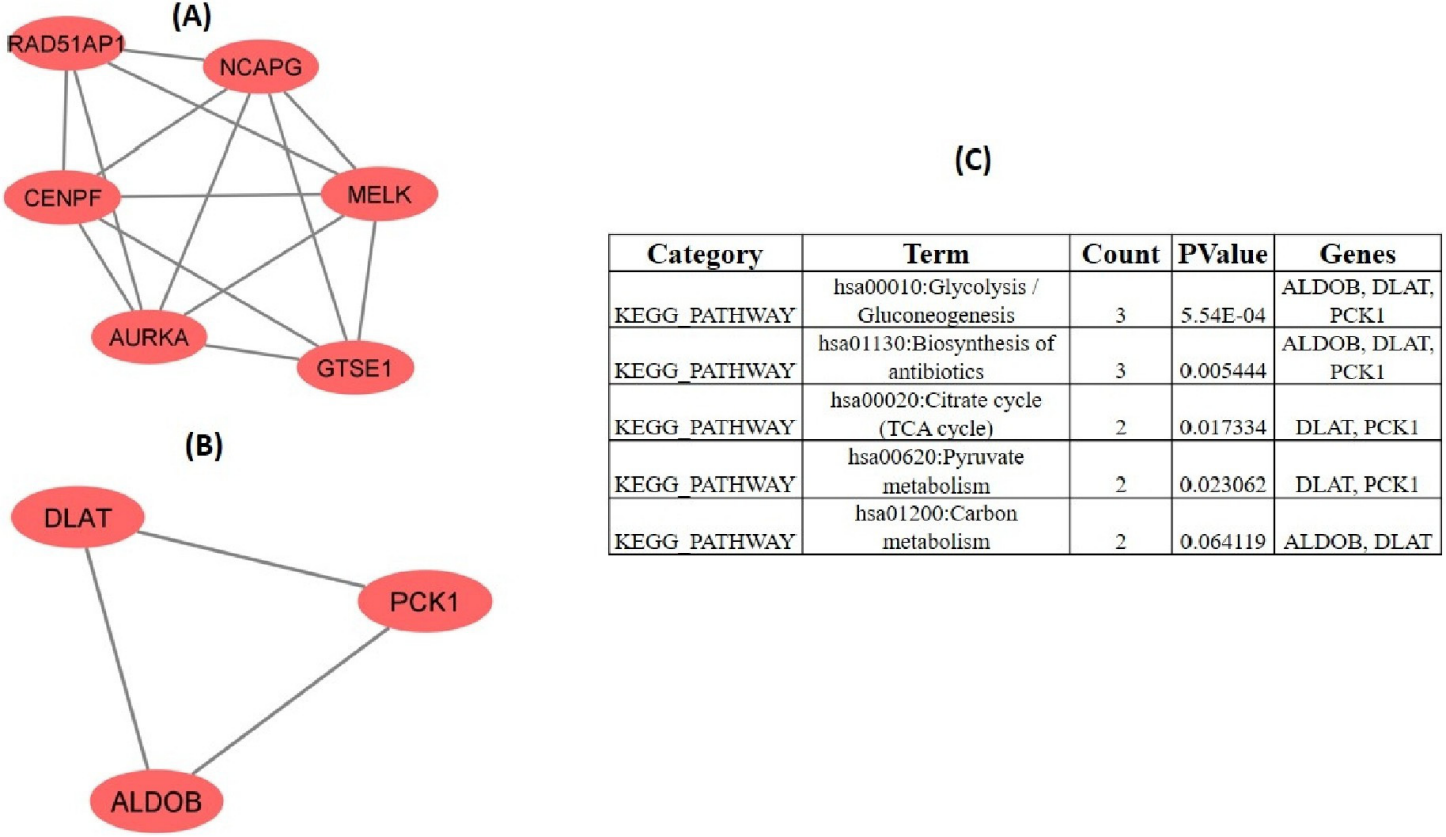
2 modules were selected having cutoff criteria node ≥ 3 and the score is ≥ 3.(A) First module constructed from MCDOE comprised of 6 genes(B) Second module constructed from MCDOE comprised of 3 genes(D) Pathways associated with the each gene in 2 modules.

PPI network of DEGs obtained from STRING (Fig. 1) were subjected to the MCODE plugin of cytoscape in order to analyze the significant modules. From these modules, the top two functional clusters of modules were selected based on the cutoff criteria of node ≥ 3 and the score is ≥ 3 (Table 3). KEGG pathway analysis of the selected modules revealed that the genes glycolysis/gluconeogenesis, biosynthesis of antibiotics, citrate cycle, pyruvate metabolism, and carbon metabolism (Fig. 2).

**Table 3:** Top 2 modules were selected having cutoff criteria node ≥ 3 and the score is ≥ 3.

**Clusters**	**Score**	**Nodes**	**Edges**	**Nodes IDs**
1	5.600	6	14	AURKA, GTSE1, RAD51AP1, NCAPG, MELK, CENPF
2	3.000	3	3	ALDOB, PCK1, DLAT

### 3.4 Selection of hub genes

**Fig. 3:**
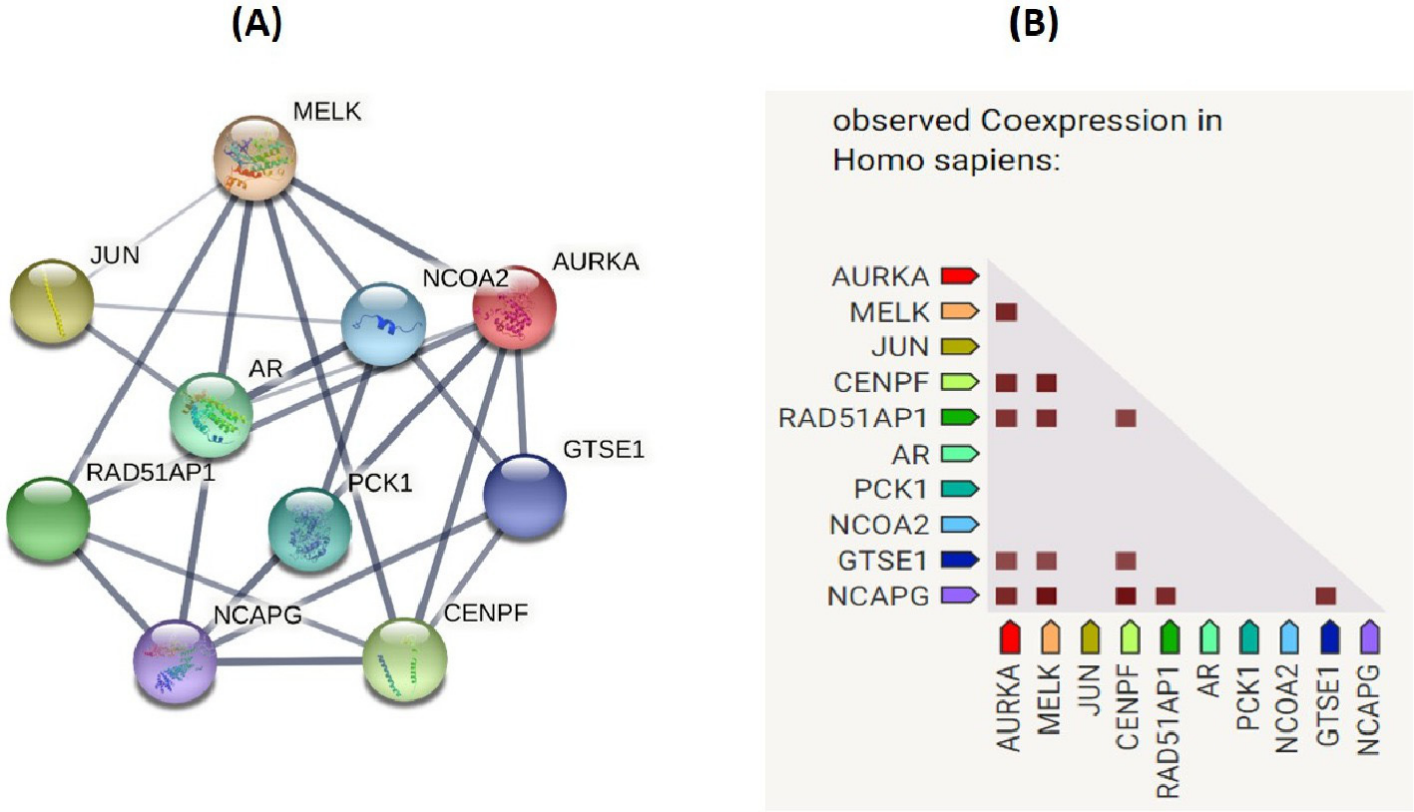
(A) Construction of PPI network among 10 hub genes (C) Coexpression analysis of 10 hub genes using STRING.

**Fig. 4:**
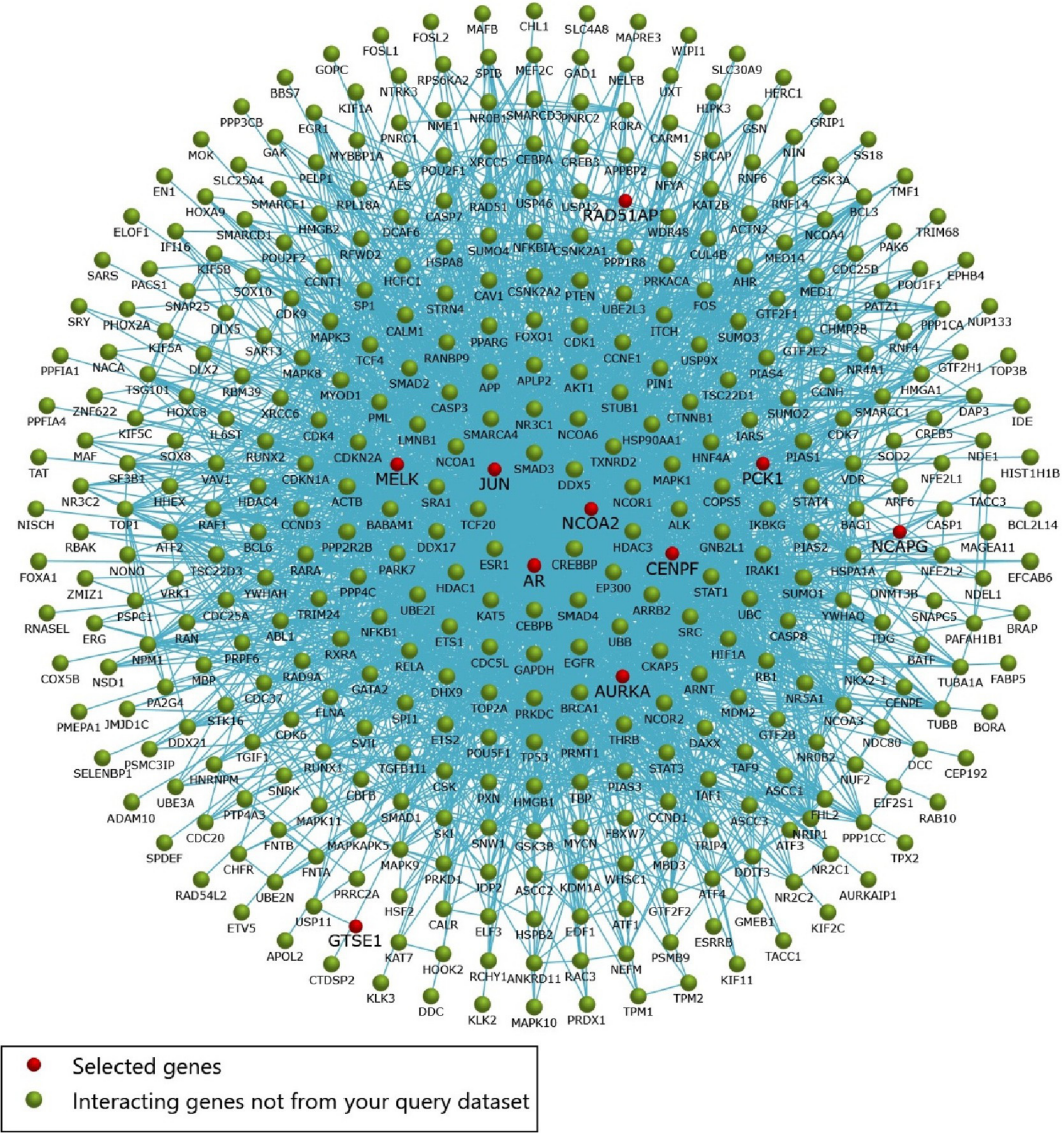
Interaction network of hub genes to their related neighboring genes.

Using 12 methods available in the cytoHubba, the topmost ten genes were selected and ranked by degree method. These ten genes named AURKA, JUN, AR, MELK, NCOA2, CENPF, NCAPG, PCK1, RAD51AP1, GTSE1 were considered as the hub genes (Fig. 3). Moreover, the interaction network of hub genes to their related neighboring genes is shown in Fig. 4.

### 3.5 TF-gene interaction network

**Fig. 5:**
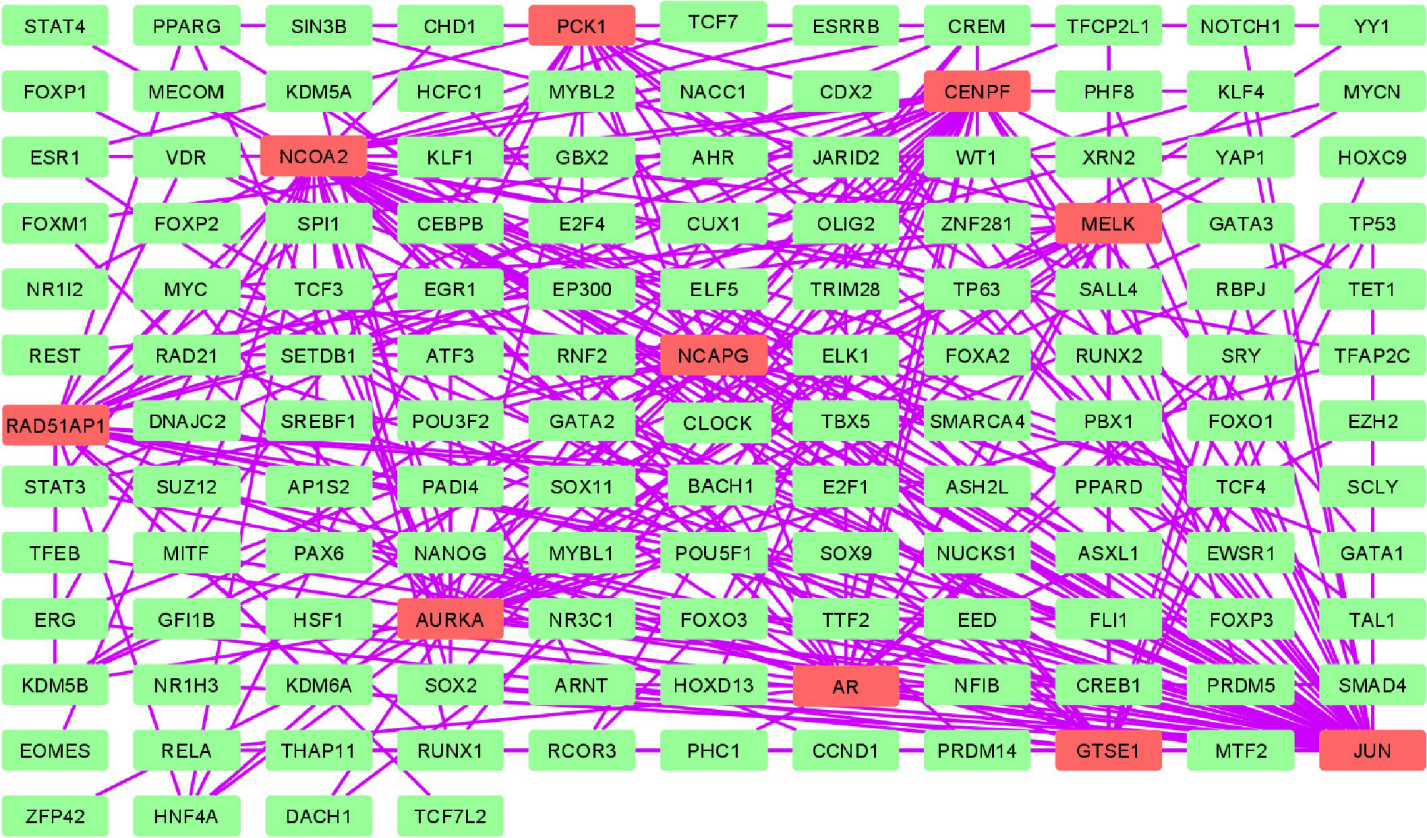
Construction of TF-hub genes interaction network from cytoscape. Red rectangles in network represent the hub genes while the green rectangles in network represent TF followed by arrows which shows the interaction among TF and hub genes.

A total of 136 nodes and 266 edges of the 10 hub genes were examined from Networkanalyst software. Subsequently, the resulted network was imported to Cytoscape for visualization of interaction among TFs and hub genes (Fig. 5). The top ranked TFs were MYC, KDM5B, STAT3, TCF4, CREM, NANOG, SOX2, HNF4A, FLI1, and ASH2L Based on the results, we found that degree level of JUN was very high as it was coregulated by 27 TFs.

### 3.6 Drug-gene interaction

**Fig. 6:**
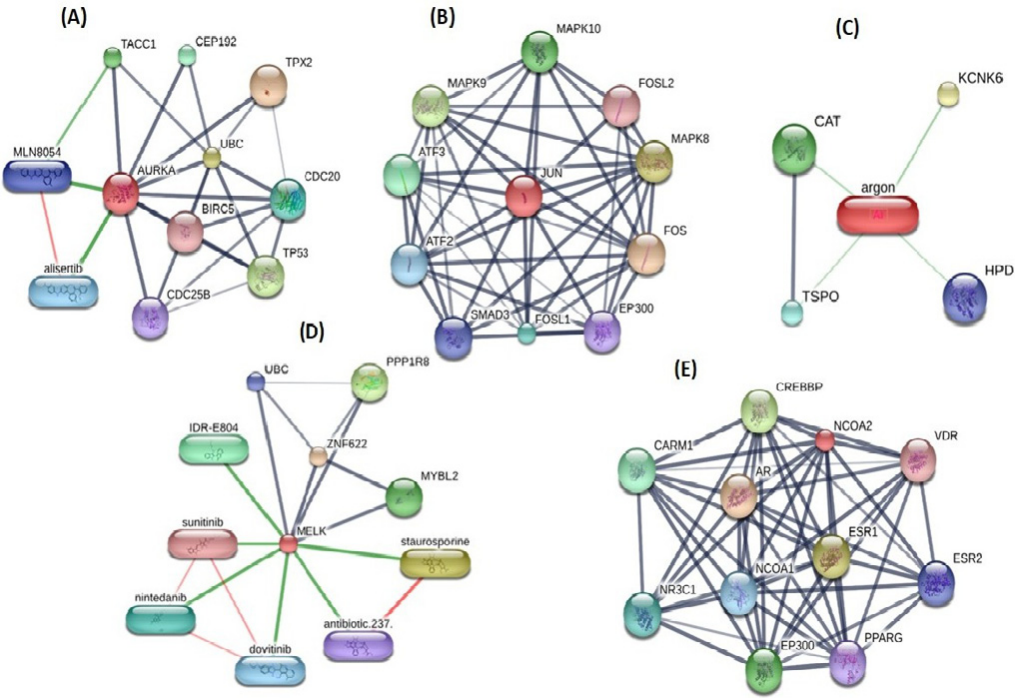
(A) Targetable AURKA subnetwork (B) Targetable JUN subnetwork (C) Targetable AR subnetwork (D) Targetable MELK subnetwork (B) Targetable NCOA2 subnetwork.

A total of 47 drugs were explored using DGIdb that might have potential to treat affected patient. AURKA, JUN, AR, MELK, and NCOA2 were chosen as possible targets of 47 drugs based on DrugBank source (Table 4). Furthermore, using STITCH tool, downstream interaction networks of AURKA, JUN, AR, MELK, and NCOA2 were generated (Fig. 6).

**Table 4:** List of FDA approved drugs.

**Genes**	**Drug**	**Interaction_types**	**Sources**	**Pmids**
AURKA	FOSTAMATINIB	inhibitor	DrugBank	26,516,587
JUN	VINBLASTINE	other/unknown	DrugBank	16555127|15498923|12907245|17126817|16111654
JUN	ADAPALENE	antagonist	DrugBank	26947815|15727806
JUN	IRBESARTAN	other/unknown	DrugBank	15133856|15210574
AR	PRASTERONE	agonist	DrugBank	15,994,348
AR	TESTOSTERONE PROPIONATE	agonist	DrugBank	17086931|17084172|17128417|17322500|17202804|12604714
AR	BICALUTAMIDE	antagonist	DrugBank	12517791|20381361|26000489|10754148|23017882|21050768|29211833|22175694|10500149|11752352|10076535|11931851
AR	ENZALUTAMIDE	inhibitor|antagonist	DrugBank	23779130|25184630|25634130
AR	FLUOXYMESTERONE	agonist	DrugBank	8119180|6439037|2521824|17023534|11752352|10077001
AR	DAROLUTAMIDE	inhibitor|antagonist	DrugBank	31571095|30197098|28801852
AR	DANAZOL	agonist	DrugBank	9593936|2404115|2486535|10882672|18061638
AR	SPIRONOLACTONE	antagonist	DrugBank	18,819,053
AR	OXYMETHOLONE	agonist|activator	DrugBank	16,633,980
AR	FLUTAMIDE	antagonist	DrugBank	11162924|26000489|10822172|10500149|10752671|12231070|11752352|10879806
AR	NILUTAMIDE	antagonist	DrugBank	12497018|26000489|3320565|16986000|3071951|20541672|11752352|12497048|12496872|6374639
AR	NANDROLONE DECANOATE	agonist	DrugBank	18,809,391
AR	APALUTAMIDE	antagonist	DrugBank	23779130|22266222
AR	NORGESTREL	agonist	DrugBank	3,139,361
AR	DROMOSTANOLONE PROPIONATE	agonist	DrugBank	15351799|3758193|11752352
AR	METHYLTESTOSTERONE	agonist	DrugBank	17086931|17084172|17128417|17322500|17202804|11752352
AR	KETOCONAZOLE	binder	DrugBank	1,526,623
AR	OXANDROLONE	agonist	DrugBank	15219414|17364004|11752352|20230007|11392377
AR	TESTOSTERONE	agonist	DrugBank	17086931|17084172|17128417|17322500|17202804|11752352
AR	DROSPIRENONE	antagonist	DrugBank|	15134826|7625729|11024226
AR	NANDROLONE PHENPROPIONATE	agonist	DrugBank	12760377|17405825|14761877|11752352|14619588|14663936
AR	STANOZOLOL	agonist	DrugBank	16159155|6539197|12589933
AR	LEVONORGESTREL	agonist|binder	DrugBank	14672731|19836445|3139361|19833195
AR	NORELGESTROMIN	partial agonist	DrugBank	15,625,768
AR	MITOTANE	antagonist	DrugBank	9,705,896
AR	GESTRINONE	antagonist	DrugBank	
AR	DIENOGEST	antagonist	DrugBank	18,061,638
AR	OXYBENZONE	antagonist	DrugBank	15537743|15950433
AR	PROGESTERONE	potentiator|agonist	DrugBank	19111796|10509795|1397870
AR	NORETHINDRONE	agonist	DrugBank	15,063,480
AR	NORGESTIMATE	partial agonist	DrugBank	15,625,768
AR	SEGESTERONE ACETATE	agonist	DrugBank	11,108,869
AR	TRICLOSAN		DrugBank	20,943,248
AR	NORETHYNODREL		DrugBank	20,438,827
AR	TAMOXIFEN		DrugBank	14,751,673
AR	FLUFENAMIC ACID		DrugBank	17911242|10592235
AR	FLUPHENAZINE		DrugBank	17,606,915
AR	ESTRONE		DrugBank	12,676,605
AR	ULIPRISTAL		DrugBank	23,437,846
AR	ACETOPHENAZINE		DrugBank	17,606,915
AR	PERICIAZINE		DrugBank	17,606,915
MELK	FOSTAMATINIB	inhibitor	DrugBank	26,516,587
NCOA2	ESTRADIOL BENZOATE		DrugBank	15,123,288

## 4 Discussion

T2DM is conventionally demarcated as an endemic disease worldwide. Slew of studies has made it clear that T2DM is major risk factor for the development of HCC (Huang et al., 2007, Wang et al., 2009, Hsiang et al., 2015). The chances of getting HCC is particularly higher nearly 2 to 3 times in patient suffering from T2DM (Su et al., 2015). Despite numerous studies on the association between HCC and T2DM, the underlying mechanisms behind the development of HCC in patient suffering from T2DM is still lacking (Mukherjee et al., 2015). There has been substantial heterogeneity regarding various cases that leads the earlier detection, a thought-provoking question. The current work planned to identify the disease related functional genes involved in the progression of HCC in T2DM patient. This whole research revolves around the analysis of gene ontology, gene enrichment pathways, PPI, hub genes, and drug-gene interaction. Four different datasets were analyzed using integrated bioinformatics analysis. Through KEGG pathway analysis, the DEGs were significantly found to be enriched in the glycolysis/gluconeogenesis, biosynthesis of antibiotics, and pathways in cancer. Our functional annotation of target genes might be helpful in understanding this targeted slicing on the development of both disease at once. In the current work, total 10 genes were found to be altered in patients involving AURKA, JUN, AR, MELK, NCOA2, CENPF, NCAPG, PCK1, RAD51AP1, and GTSE1. Hence, it represents that these genes play important role in the development of HCC in T2DM patients. Furthermore, 47 drugs of AURKA, JUN, AR, MELK, and NCOA2 were found having therapeutic potential to treat HCC patients with T2DM.

JUN is an oncogene, encodes for c-jun protein. Multiple studies of evidence on JUN gene regarding their contribution in HCC has made it clear that variation in JUN concerned with the development of HCC (Endo et al., 2009, Yuen et al., 2001). TFs analysis of hub genes in the current analysis revealed that degree level of JUN was very high, hence TFs of JUN might play important roles in the development of HCC in persons with T2DM. All these evidences might prove fruitful to combat the disease condition by preventing HCC from becoming malignant. These findings are further strengthened by KEGG pathway analysis which revealed that JUN genes contribute to the multiple pathways of cancer. By targeting JUN, the pathogenic mechanisms of HCC in T2DM patients can be controlled, hence might serve as molecular biomarker for the diagnosis and treatment.

AURKA is a mitotic serine/threonine kinase, crucial for the cell cycle progression. During the last ten years, slew of studies has made it clear that alteration in AURKA gene encourages the development of HCC hence might serve as potential diagnostic biomarker (Wang et al., 2018). Moreover, many bioinformatics related studies on HCC has also been enlisted the AURKA as key genes involved in the progression of HCC (Zhou et al., 2018). Although, involvement of AURKA genes has not been discovered in case with T2DM. Hence all these evidences provide a precious clue that upregulation of AURKA might control the intricate molecular mechanism behind the pathogenesis and development of HCC in individuals with T2DM and might serve as biological marker to detect both diseases at early stages.

MELK is considered as key member of AMPK family, and a therapeutic target for multiple type of cancers. Numerous studies regarding the development of HCC provide evidence that variation in expression level of MELK is concerned with the development of HCC (Jiang and Zhang, 2013, Xia et al., 2016). Hence by considering MELK as molecular biomarker, the diagnosis and treatment of HCC in T2DM patients might become an easy task. Considering our analysis, we propose that upregulation of MELK might induce HCC in T2DM patients.

Identification of aberrant pathways in affected patient might help to identify the molecular mechanism underlying and to uncover more enthralling and promising molecular candidates with effective diagnostic and prognostic value. It is noteworthy that KEGG pathway analysis of JUN, AR revealed that these gene are key members of the pathways in which small disruption will unfortunately leads to cancer. These findings shed light on the pathogenesis of both diseases and facilitate the development of personalized treatment. The disturbed pathways identified using integrated bioinformatics analysis may have important role to play in the pathogenesis of both diseases. Additional studied is required to investigate the molecular mechanisms behind these aberrant pathways and development of HCC in individuals with T2DM.

In conclusion, this research discerned hub genes as key biological marker and their associated pathways involved in the development of both diseases. In near future, further study and clinical trials are required for the identification of genes and small drug like molecule having effective diagnostic and prognostic value, respectively. This research relies on various freely available databases to shed light on pathogenesis and treatment and both diseases at once. *In vivo *and *in vitro* investigation of gene and pathway interaction is essential to delineate the specific roles of the identified genes, which may help to confirm gene functions and reveal the mechanisms underlying the development of both diseases. Additional experimental research on these hub genes lead to increase our knowledge to fight against HCV-HCC in patients with T2DM in future by means of novel therapeutic approaches.

## 5 Conclusion

In the present work, a new mechanism was proposed which explain that progression in pathogenesis of both diseases might due to the genes that disturbs the pathways which ultimately leads to disease condition. AURKA, JUN, AR, MELK, and NCOA2 has not been previously reported to be related to HCV-HCC in individuals with T2DM, hence these genes might act as potential biomarkers for diagnosis of both diseases at early stage. Our findings reveal that hub genes cause disruption in cellular pathways which unfortunately make the disease condition much worse. Our research will serve as significant pioneer for the researchers who wants to identify the associated pathways involved in the development and pathogenesis of both diseases. Based on the hub genes, experimental models may be designed in terms for the detection of pathogenesis, evaluation of risk, and in determining the targeted therapies.

## Ethics approval

Not Applicable.

## Consent to participate

All authors consent to participate in this manuscript.

## Consent for publication

All authors consent to publish this manuscript in Saudi journal of Biological Science.

## Availability of data and material

Data will be available on request to the corresponding or first author.

## Code availability

Not Applicable.

## Declaration of Competing Interest

The authors declare that they have no known competing financial interests or personal relationships that could have appeared to influence the work reported in this paper.
